# Targeting Cysteine Oxidation in Thrombotic Disorders

**DOI:** 10.3390/antiox13010083

**Published:** 2024-01-09

**Authors:** Moua Yang, Roy L. Silverstein

**Affiliations:** 1 Division of Hemostasis and Thrombosis, Beth Israel Deaconess Medical Center, Harvard Medical School, 3 Blackfan Circle, CLS-924, Boston, MA 02115, USA; 2 Department of Medicine, Medical College of Wisconsin, Hub 8745, 8701 W Watertown Plank Rd., Milwaukee, WI 53226, USA; 3 Versiti Blood Research Institute, Milwaukee, WI 53226, USA

**Keywords:** oxidative stress, cysteine, CD36, protein disulfide isomerase, thrombosis, platelets, nucleophiles

## Abstract

Oxidative stress increases the risk for clinically significant thrombotic events, yet the mechanisms by which oxidants become prothrombotic are unclear. In this review, we provide an overview of cysteine reactivity and oxidation. We then highlight recent findings on cysteine oxidation events in oxidative stress-related thrombosis. Special emphasis is on the signaling pathway induced by a platelet membrane protein, CD36, in dyslipidemia, and by protein disulfide isomerase (PDI), a member of the thiol oxidoreductase family of proteins. Antioxidative and chemical biology approaches to target cysteine are discussed. Lastly, the knowledge gaps in the field are highlighted as they relate to understanding how oxidative cysteine modification might be targeted to limit thrombosis.

## 1. Introduction

Many pathologic conditions are associated with oxidative stress and have increased risk for clinically significant thrombotic events. These conditions include, but are not limited to, disorders of metabolism (e.g., dyslipidemia [1,2], diabetes mellitus [3], and obesity [4]), chronic systemic inflammation [1], aging [5], cancer [6], infection [7], and blood disorders including hemoglobinopathy [8,9], and antiphospholipid syndrome [10]. Clinically significant thrombotic events promote organ dysfunction, organ failure, and sometimes death [11]. Thrombus formation is a complex, multifaceted process, and oxidative events initiated by reactive species of oxygen, nitrogen, sulfur, and carbon play important roles. These oxidative species induce modifications of cellular constitutions, including lipids and proteins [12]. Oxidative modifications of proteins do not induce clotting on their own, nor are they the final effectors of clotting, but like phosphorylation events that sensitize downstream signaling pathways, oxidative modifications may enhance or limit thrombosis [13] and thus provide additional layers of control of the thrombotic process.

Essential to oxidative post-translational modification of proteins are the reactive species themselves. The chemical reduction or oxidation of molecular oxygen generates reactive oxygen species (ROS), and a detailed review of specific species and their generation mechanisms is provided in a recent review [14]. It is the two-electron oxidants, such as hydrogen peroxide, lipid hydroperoxide, and peroxynitrite, that oxidize thiol side chains of cysteines [15]. Other amino acids are also susceptible to oxidation by ROS; for example, peroxynitrite formed from interaction of superoxide radical and nitric oxide oxidizes tyrosine to form nitrotyrosine [16]. Hypochlorous acid (HOCl), generated by myeloperoxidases in white blood cells, oxidizes methionine and halogenates tyrosine to generate methionine sulfoxide [17] and halo-tyrosine [18], respectively. In the context of thrombosis and hemostasis, we recently provided a review on oxidative cysteine and methionine modification [13].

As we delve deeper into the redox biology of thrombosis, fundamental knowledge gaps remain pertinent to understanding how the thrombotic machinery is transformed by redox cues from a protective homeostatic process to a pathological vaso-occlusive thrombotic process. Essential to this knowledge gap is the question of whether it is one protein or a network of proteins that become oxidized or reduced to promote pathologic thrombosis. Also, could specific enzymes sense these redox cues and transform the promiscuous electrophilic nature of oxidants to an enzyme-driven process allowing for specificity in thrombotic signaling pathways? And lastly, could these oxidative events be targeted therapeutically to decrease pathologic thrombus without compromising hemostasis? In this review, we discuss cysteine biochemistry relating to its reactivity, the different types of oxidative cysteine modification, and recent advances in cysteine sulfenylation in oxidative stress related thrombosis. We also discuss potential therapeutic avenues to oxidative cysteine modification rooted in evidence from the use of a nucleophilic fragment library designed to target oxidized thiols.

## 2. The Biochemistry of Cysteines

### 2.1. Cysteine Reactivity: Not All Are Created Equal

Because of its unique redox properties, cysteine plays diverse roles in protein structure and function. Evolutionarily, cysteines are found at both highly conserved and non-conserved sites, and not surprisingly, mutations at these sites often result in pathologic conditions [19]. Cysteine pairs within proteins can form disulfide bonds, thereby contributing importantly to secondary and higher order structure. These residues are often buried from solvent exposure [20]. Cysteines can also be in their free thiol state, where they are exposed to solvent and play non-structural roles, including regulation of enzyme activity (e.g., in kinases) [21,22], metal binding (e.g., Zn-finger transcriptional factors) [23], catalytic redox reactions (e.g., thiol isomerases as described in Section 3) [24,25], and catalytic nucleophilic reactions (e.g., caspases and phosphatases) [26]. The nucleophilicity of the thiol is determined by the stabilization of the negatively charged thiolate [27], a physicochemical property of the sulfur atom.

Deprotonation of the cysteine thiol is a critical step in generating the nucleophilic thiolate and depends on the acid dissociation constant or p*K_a_* of the cysteine and the local pH of the environment [28]. If p*K_a_* is lower than the pH, the fraction of cysteine in the deprotonated form is higher. At physiologic pH ~7.4, the cysteines with p*K_a_* of less than 7.4 should have a higher fraction in the thiolate form and are thus more “reactive”. However, it should be noted that the p*K_a_* of the thiol group of free cysteine is ~8.3 and can range substantially (from 3.5 to 13 depending on the microenvironment) [29,30]. The pH of the local environment is also not uniform [30]; lysosomes, for example, have much lower pH (pH 4–5) relative to the cytosol (pH 6.8–7.4) and mitochondria (pH~8.0 within mitochondrial matrix) [31]. As the p*K_a_* is linked to the pH of the aqueous environment, solvent accessibility (e.g., buried vs. exposed) influences cysteine reactivity. Additional factors that influence thiolate formation include proximal structural elements of the protein that can stabilize the negative charge [32]. Hydrogen bond donors, such as the hydroxyl group of threonines as in thioredoxins [33], have a partial positive charge from the hydrogen allowing for stability in the thiolate through bonding with the sulfur atom [29]. Electropositive (basic) amino acids including arginine and histidine afford additional ionic interaction with the negative charge of the sulfur and stabilize the thiolate for redox events [34]. Similarly, positively charged macrodipoles of alpha helices afford electrostatic interactions with the thiolate for stability [35].

Cysteine reactivity is also controlled by the redox potential of the thiol. Electrons are poised to proceed from the more electronegative to the more electropositive redox potential, with the potential ranging substantially in biologic systems (from −480 to −80 mV) [36]. In an isolated system, these potentials could be calculated for a thiol using different pairs of redox couplers (e.g., reduced glutathione (GSH) with oxidized glutathione (GSSG); reduced dithiothreitol with oxidized dithiothreitol; cysteines and cystines); however, the dynamics of redox potential within a biological system are quite complex and difficult to calculate owing to the different redox couplers in any given location [30]. This suggests that cysteines could be more reactive within different sub-compartments of the cell, with a classic example being the endoplasmic reticulum where the environment is more oxidizing to facilitate oxidative protein folding.

Lastly, an environmental factor that influences cysteine reactivity is proximity to the source of oxidants. Many sources within a cell promote electron leakages (and transfers) that could generate reactive oxygen species [37]. A protein that is closer to a source of oxidant is more likely to encounter the reactive species prior to the oxidant degrading through an efficient antioxidative mechanism or through competing oxidizing targets. Proteins further away from a source of oxidant are protected as the oxidants have less of a chance to reach the target. Evidence connecting proximity of a protein to an oxidant source and oxidation of that protein were demonstrated in studies of an epidermal carcinoma cell line [38]. In these cells, Epidermal Growth Factor (EGF) binding to its receptor (EGFR) [38,39] promotes receptor dimerization and activation, allowing for downstream signaling for proliferation, differentiation, growth, and survival [40]. The proximity of EGFR to NADPH oxidase was shown to promote cysteine oxidation of the receptor, enhancing kinase activity and downstream signaling.

### 2.2. Cysteine Oxoforms

**Figure 1:**
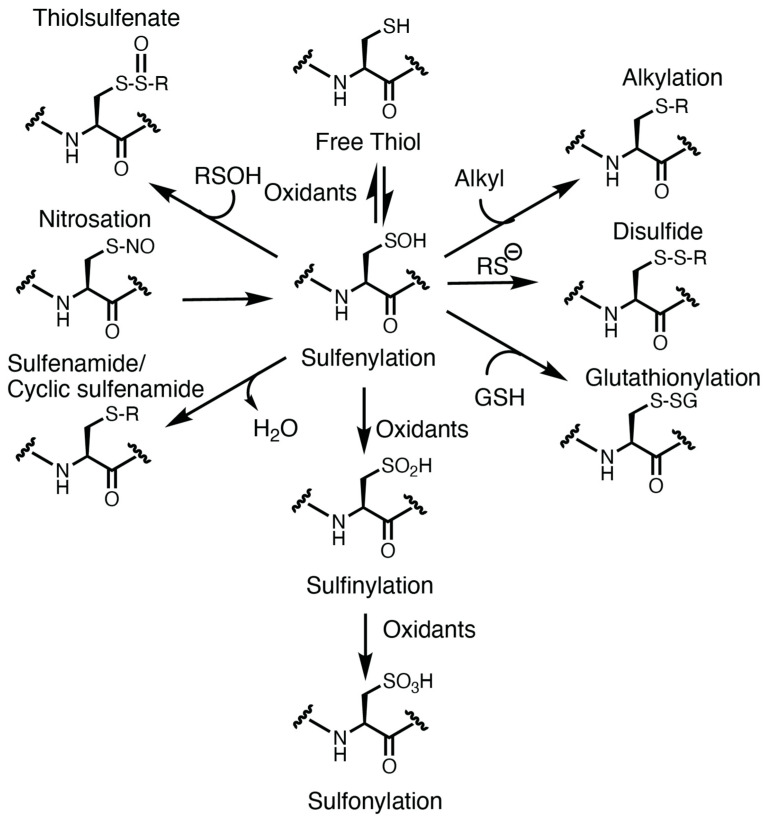
Oxidative modification of cysteine. The cysteine thiolate is nucleophilic and susceptible to oxidation. Oxidation of the thiol generates a transient and labile modification: the sulfenic acid (sulfenylation). Sulfur oxidation by nitric oxide or nitrosothiols could also lead to sulfenic acid formation. Sulfenylation is a cysteine oxoform that is at the crossroad of further oxidative cysteine modification, including sulfinylation, sulfonylation, glutathionylation, disulfide, alkylation, thiolsulfenate, and sulfenamides.

Oxidation of the cysteine thiolate generates many sulfur oxoforms. A depiction of these oxoforms is presented in Figure 1. The sulfur atom attains valences between −2 and +6 oxidation states [15]. Oxidation of the thiolate by peroxide generates a sulfenic acid, which is an intermediate oxoform that is labile and susceptible to convert to other oxoforms [15]. Further oxidation of the sulfenic acid could generate sulfinic and sulfonic acid. Oxidation of the sulfinic acid is reversible with sulfinic acid reductase [41], whereas the sulfonic acid is currently believed to be irreversible. Sulfenic acids could also lead to the formation of other oxidative cysteine modifications, including cysteine glutathionylation (by reaction with glutathione), sulfenamides (reacting with an amine) [42], thiolsulfenates (reacting with a nearby sulfenic acid) [15], and disulfides [15,43]. In some instances, other cysteine oxoforms could be converted over to a sulfenic acid (as is the case for nitrosothiols) [15]. These oxoforms emphasize the significance of the sulfenic acid as a “gateway” modification that may be able to be targeted pharmacologically.

## 3. Oxidative Cysteine Modification in Oxidative Stress-Driven Thrombosis

### 3.1. Thrombosis Is a Multifaceted Process

**Figure 2:**
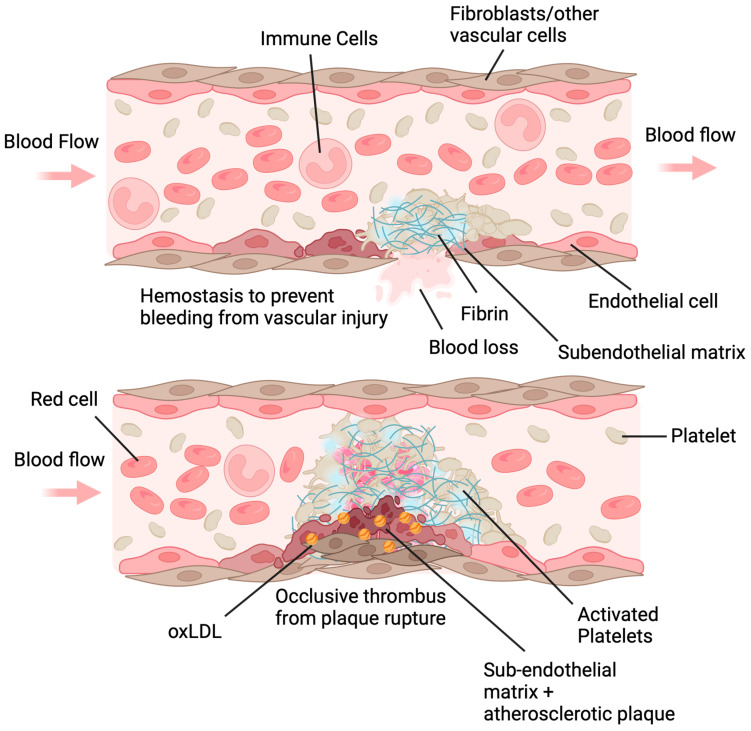
Hemostasis and thrombosis. (**Top**) In healthy states, a physical injury to a blood vessel causes extrusion of the blood, but the blood loss is limited by formation of a hemostatic plug generated by platelet adhesion and aggregation at the site of injury. Fibrin deposition generated from activation of the coagulation serine protease cascade at the site of injury creates a proteinaceous network that stabilizes the plug. (**Bottom**) In pathological conditions associated with oxidative stress, the normal hemostatic response is amplified and can lead to formation of an occlusive thrombus at the site of a minor injury, such as occurs in the setting of a small rupture of atherosclerotic plaque. Created with BioRender.com and accessed on 4 January 2024. oxLDL, oxidized low-density lipoprotein particle.

Hemostasis, the physiological system that maintains blood fluidity and vascular integrity, is a highly regulated, tightly balanced process. The ability to prevent excessive bleeding after vascular injury involves many components of the vasculature, including cells and factors that are derived from both hematopoietic and non-hematopoietic lineages [44]. Endothelial cells that line the lumen of the vessel are quiescent and express antithrombotic entities [45], including prostacyclin [46] and nitric oxide [47], which are released to dampen platelet responses and inhibit thrombosis. These factors activate adenylyl and guanylyl cyclases within platelets, generating cAMP [48] and cGMP, respectively [47]. These cyclic nucleotides are secondary messengers promoting a cascade of phosphorylation events driven by protein kinase A and protein kinase G that limit platelet activation [48]. Cell surface expression of anticoagulant molecules including heparan sulfate proteoglycan and thrombomodulin also limit the thrombotic process [45] by interfering with thrombin generation and fibrin formation. Antiplatelet ectonucleotidases are associated with endothelial cells, including CD39 and CD73, and prevent nucleotide induced activation of platelets [49]. When the continuity of the endothelial layer is disrupted by injury (Figure 2, top), the underlining extracellular matrix is exposed to flowing blood leading to platelet recruitment, adhesion, and aggregation resulting in formation of the primary hemostatic plug [50]. In addition, exposure of the transmembrane protein tissue factor to blood at the site of injury activates the coagulation cascade through the prothrombinase and tenase serine protease complexes. These complexes ultimately lead to the generation of thrombin, a protease that cleaves plasma soluble fibrinogen to insoluble fibrin monomers which polymerize to form a fibrin mesh, stabilizing the clot [44]. Exposed phosphatidylserine on activated platelets further amplifies thrombin-generation potential by serving as a cofactor for the prothrombinase and tenase complexes. Dysregulation of this process by oxidants could contribute to the pro-thrombotic state associated with metabolic syndromes, chronic inflammation, and other pathogenic conditions where thrombus formation could occlude the lumen of the vasculature (Figure 2, bottom).

### 3.2. CD36 Links Oxidative Stress to Prothrombotic Cysteine Oxidative Signaling

Evidence for oxidative stress-driven thrombosis is derived from multiple independent studies using targeted interruption of genes involved in oxidant regulation [51,52,53], pharmacologic inhibition of oxidant sources [54,55], chemical degraders or generators of oxidants [56,57], cellular assays that involve oxidants (e.g., platelet activation and endothelial cell-mediated procoagulant activity) [58,59,60], and animal models of thrombosis [57,61]. We focus on atherothrombosis, which is arterial thrombosis in the context of atherosclerosis, based on evidence from our lab and others that oxidative stress is prominent in dyslipidemia and other chronic atherogenic states and that redox cues in these settings are converted to a pro-thrombotic response [2,62,63].

Dyslipidemia refers to abnormal levels and/or composition of lipids in the circulation that are mainly carried by lipoprotein particles [64,65]. In atherothrombosis, dyslipidemia links atherosclerotic plaque development, plaque instability, and ultimately plaque rupture with subsequent thrombosis [65]. Among the different types of lipoproteins present in the circulation are high-density lipoprotein (HDL) and low-density lipoprotein (LDL) [65]. These particles are susceptible to oxidation during the inflammatory process of plaque development, and oxidation generates oxidized lipid moieties on the particles that are recognized by specific proteins of the innate immune system [66,67]. Oxidation of LDL (oxLDL) creates a high-affinity ligand for the scavenger receptor CD36 [68,69], a transmembrane glycoprotein of the innate immune system that is expressed on multiple vascular cells and was first identified as the fourth-largest major membrane protein on platelets [70]. Recognition of oxLDL by platelet CD36 promotes multiple signaling events that link to desensitization of platelet inhibitory pathways [54,71,72] or sensitization of platelet activation pathways [55,71,73]. A comprehensive understanding of platelet CD36 signaling is still an active area of research and has recently been reviewed [71].

**Figure 3:**
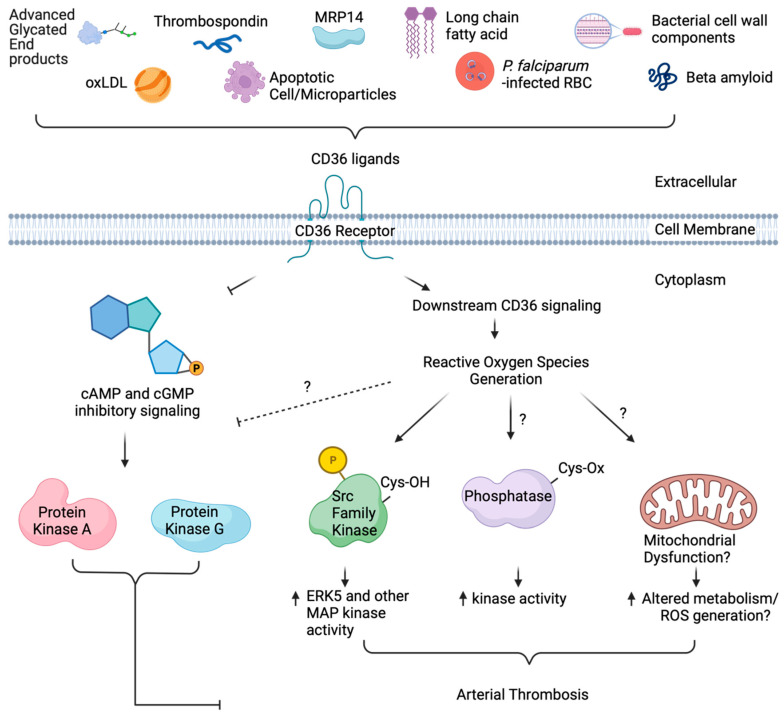
Platelet CD36 redox signaling in arterial thrombosis. Platelet CD36 has multiple ligands including oxidized lipids in lipoprotein particles, advanced glycated end products, thrombospondin, long chain fatty acids, *P. falciparum*-infected red cells, myeloid-related protein 14 (S100A9; MRP14), components of bacterial cell wall, apoptotic cells and microparticles, and beta amyloid. The best-characterized CD36 ligand is oxidized phospholipid present in oxLDL. Recognition of these ligands by CD36 at the surface of the cell promotes two downstream signaling pathway within platelets. One is through desensitization of the cAMP and cGMP signaling pathway that are the major inhibitory pathways within platelets, and the other is through downstream ROS signaling. The effects of ROS are multifaceted and include oxidative cysteine modification of Src family tyrosine kinases to enhance kinase activity and increase the activation of big MAP kinase ERK5 for a pro-aggregatory and pro-coagulant platelet phenotype. In macrophages and endothelial cells, CD36-mediated ROS generation inactivates specific mixed phosphatases including SHP-1 and SHP-2, resulting in prolonged activation of downstream kinases. In addition, CD36 alters mitochondrial metabolism within macrophages into a pro-inflammatory, pro-atherogenic, and ROS-generating organelle. We hypothesize similar mechanisms within platelets for phosphatase inactivation and mitochondrial dysfunction to enhance prothrombotic kinase activity and oxidative stress in dyslipidemia. It is also possible that ROS generated through CD36 activation participates in desensitizing the inhibitory pathways through oxidative cysteine modification. Created with BioRender.com and accessed on 4 January 2024. ?, hypothesized mechanism that requires investigation; ↑ with no faded tail, upregulates or increases arrow; ↓ with faded tail, cellular signaling arrow; ⊥ with faded tail, inhibitory signaling or inhibits.

Generation of ROS in platelets by CD36 signaling contributes to its prothrombotic signaling (Figure 3). In addition to oxidized lipids, CD36 has many ligands including advanced glycated end products (AGEs) [74], apoptotic cells [75] and microparticles [76,77], thrombospondins and other proteins containing the thrombospondin type 1 repeat domain [78,79], long-chain fatty acids [80], *Plasmodium falciparum*-infected red cells [81], components of bacteria cell wall [82,83], beta amyloid [84], and the myeloid-related protein 14 (or S100A9) protein [85]. We and others have found that CD36 recognition of oxLDL promotes recruitment and activation of the Src family tyrosine kinases, Fyn and Lyn as signaling transducers [73]. Src kinases phosphorylate many downstream components [86], including a pathway that leads to the activation of the oxidant-sensitive mitogen-activated protein (MAP) kinase, Big MAP kinase/extracellular signal-regulated kinase 5 (ERK5) [55]. Src kinase signaling also cascades to the activation of the subunits of reduced nicotinamide adenine dinucleotide phosphate (NADPH) oxidase that promotes electron transfer from NADPH to molecular oxygen [87]. Aside from the mitochondrial respiratory chain, the electron transferring event induced by NADPH oxidase is a major pathway for generating superoxide radical anion [14,88]. Superoxide anion is an unstable ROS, however, and does not directly react with free thiols. In addition, superoxide anion also does not traverse the cell membrane due to the nature of its charge. Superoxide anion rapidly dismutates both spontaneously (10^5^ M^−1^ s^−1^) and catalytically via superoxide dismutase (2 × 10^9^ M^−1^ s^−1^) [89], converting the oxidant to the more stable hydrogen peroxide that is electrophilic, able to diffuse across the cellular membrane, and react with free thiols.

We found that hydrogen peroxide generated from NADPH oxidase was able to sulfenylate exposed cysteines on Src kinases, converting the redox signals into auto-phosphorylation events. Although it was not directly tested, we hypothesized that Cys278 in Fyn is the key site of sulfenylation in platelets since this site is known to function as a negative regulator of kinase activity [90], and Lyn does not contain cysteine at its homologous site [91]. This supposition requires further investigation, for example, by mutating these cysteine residues to non-reactive alanines within platelets. Nonetheless, oxidation of Src family kinases promotes downstream platelet CD36 signaling, including activation of ERK5. A direct role for peroxides was further validated by inhibiting NADPH oxidase or directly scavenging hydrogen peroxide with catalase, both of which eliminated sulfenylation of Src family kinases and ERK5 activation. These approaches also prevented downstream functions of ERK5, including signaling integration with the glycoprotein VI (GPVI) pathway for apoptotic-like caspase activation and subsequent externalization of procoagulant phosphatidylserine (PSer) [92]. PSer provides the negatively charged surface for assembly of coagulation factors Xa and Va in an active prothrombinase complex, allowing for localized thrombin generation with ensuing fibrin deposition [44]. 

ERK5 also sensitizes platelets to integrin activation, lowering the threshold for platelets to aggregate for a full prothrombotic phenotype. In this context, the mechanism downstream of ERK5 is unclear. An unbiased approach to identifying phospho-targets of ERK5 by CD36 signaling would close the signaling gaps that link ERK5 to a procoagulant and pro-aggregatory platelet response. It is also unclear how Src activation by hydrogen peroxide promotes ERK5 phosphorylation. Given what is known of the mitogen-activated protein kinase kinase 5 (MEK5)-ERK5 pathway in other cell types [93], it is likely that Src family kinases directly phosphorylate upstream MAP kinase kinases upstream of ERK5, allowing for ERK5 to be activated. It could also be possible that ERK5 is directly sulfenylated. This supposition is supported by sulfenylation events with the related MAP kinase family member ERK2 [94]. ERK2 oxidation at Cys159 by peroxides influences substrate selectivity within its D-recruitment substrate recognition site (DRS). Sulfenylation at this site increases interaction between ERK2 and a model DRS-specific peptide substrate as well as the ribosomal S6 kinase A1 (RSK1) substrate. In this context, sulfenylation of ERK2 increases RSK1 kinase activity [94]. Although the presence of RSK1 and its function in platelets is yet to be studied, the oxidation of ERK2 suggests a likely sulfenylation site within ERK5 based on conservation of the cysteine and the 66% structural homology with ERK2 [95]. Sulfenylation of ERK5 could thus functionally promote platelet reactivity in dyslipidemia.

CD36 potentially links oxidants and cysteine oxidation to other cellular dysfunction that could impact the prothrombotic response during vessel injury as shown in Figure 3. Microvascular endothelial cells (MVECs) and macrophages express CD36 and ligation of the receptor with extracellular vesicles (EVs) [77], or oxLDL [96] enhances ROS generation from NADPH oxidase. ROS generation in MVECs was promoted through Fyn kinase activity, which blunts endothelial cell migration and apoptosis in response to angiogenic stimuli [77]. In macrophages, CD36-mediated ROS generation inhibits the activity of tyrosine phosphatase Src Homology Phosphatase-2 (SHP-2); inactivation of SHP-2 enhances phosphorylation of focal adhesion kinase to restrict macrophage migration while promoting macrophage spreading [96], with the net effect of trapping the cell in the neointima of a growing atherosclerotic plaque. The inactivation of SHP-2 is through oxidation of the catalytic cysteine within the active site [97]. Could similar downstream mechanisms for ROS in endothelial- and macrophage-CD36 signaling be present in platelets? While sulfenylation of platelet Src tyrosine kinases enhances kinase activity, coincident sulfenylation of the catalytic cysteines in phosphatases would serve to blunt tyrosine kinase inactivation and thereby prolong Src activity, further increasing NADPH oxidase-mediated ROS generation and platelet activation. 

Additional evidence for oxidative stress-induced thrombosis by CD36 signaling was derived from studies of mice with deletion of the gene encoding the source of the oxidant. Genetic deletion of gp91phox, the catalytic subunit of NADPH oxidase, blunts the prothrombotic effect of oxLDL on murine platelets [54]. In addition, the NADPH oxidase inhibitor peptide gp91ds-tat, or the superoxide radical scavengers TEMPO or Manganese (III) tetrakis(1-methyl-4-pyridyl)porphyrin (MnTMPyP), inhibited oxLDL-mediated platelet accumulation in an ex vivo microfluidic thrombosis model [54]. It should be emphasized that deleting gp91phox or inhibition of NADPH oxidase had limited impact on platelet activation by classic agonists, such as ADP and collagen [98], suggesting that the systems required for oxidant-induced platelet sensitization are not necessary for supporting platelet activation under “normal” hemostatic conditions. This is further supported by the lack of bleeding disorder in CD36 deficient mice or humans. It would therefore be helpful to determine whether platelet NADPH oxidase activity supports CD36-mediated thrombosis in other oxidative stress-related conditions (e.g., in diabetes [74], infection [99]).

The mitochondria are additional sources of oxidants within hematopoietic and vascular cells and are important in thrombosis and hemostasis. Based on electron micrographs, there are approximately 10–12 mitochondria per platelet [100], whereas nucleated cells may have between 80 and 2000 mitochondria [101]. The mitochondria functions to maintain the metabolic demands of the cell through combined efforts of glycolysis and oxidative phosphorylation [102]. CD36 plays an essential role in fatty acid metabolism, and its metabolic involvement is intimately linked to the mitochondrial function. Recent studies by our lab and others in macrophages revealed that CD36 recognition of oxLDL promotes mitochondrial dysfunction by inducing a metabolic shift from oxidative phosphorylation to glycolysis and disrupting electron flow through the electron transport chain to generate ROS. This in effect converts mitochondrial from an energy producing factory to a pro-atherogenic, pro-inflammatory organelle [103]. We hypothesize that similar changes may occur in platelets with CD36 signaling where lipids from oxLDL particles could similarly alter metabolic pathways, repurposing them for platelet hyperreactivity, mitochondrial ROS generation, and a prothrombotic response.

The prothrombotic properties of oxidative stress in dyslipidemia are afforded by overcoming intrinsic antioxidant defense mechanisms. Multiple genes encode these mechanisms and are largely regulated by the interaction of transcription factor nrf2 with the antioxidant regulatory element (ARE) in their promotors [104]. Peroxiredoxin-2 (prdx2) is one such gene [51], and its deletion in hyperlipidemic mice was shown to promote thrombosis in a ferric chloride injury model of carotid artery thrombosis [51]. CD36 deletion in this context rescued the phenotype, supporting a role for CD36 in preventing antioxidant defenses from blunting thrombosis. Since 15 other antioxidant genes are controlled by nrf2, it is possible that deficiencies in these other genes could also promote thrombosis in the setting of dyslipidemia [104,105]; indeed, deficiencies in glutathione peroxidase and superoxide dismutase antioxidant genes have been related to thrombosis. The selenium-based glutathione peroxidase family of antioxidant enzymes degrade hydrogen peroxide by utilizing glutathione as a reducing agent, and the glutathionylated peroxidase is recycled with thioredoxin [106]. There are eight members of the glutathione peroxidase family, with some of the members directly implicated in the thrombotic machinery. Glutathione peroxidase-1 deficiency was found to be associated with age-related thrombosis risk, and Dayal et al. showed that aged mice over-expressing glutathione peroxidase-1 were protected from accelerated venous and arterial thrombosis in large vessels. When platelets were activated by thrombin, surface expression of the activation marker integrin αIIbβ3 was higher in old mice (18 months) compared to young mice (4 months) [53]. This was accompanied by an increase in platelet-derived hydrogen peroxide, and the addition of catalase to scavenge hydrogen peroxide or apocynin to inhibit NADPH oxidase decreased the hyperreactive platelet phenotype in old mice. Mutations in the promoter region of the gene for glutathione peroxidase-3 [107], an extracellular peroxidase, are associated with increased platelet reactivity [52], oxidative damage of lipids, and increased risk for myocardial infarction and stroke [108,109] in humans.

Another example of antioxidant deficiency promoting thrombosis is with the superoxide dismutase family of enzymes [110]. In the aging model of oxidative stress, Sonkar et al. found that platelet superoxide dismutase 2 (SOD2) functions to limit thrombin generation and arterial thrombosis [111]. Mice with platelet-specific SOD2 deficiency had increased in platelet oxidant generation, sustained calcium release with cellular activation, increased platelet granule secretion, and increased phosphatidylserine externalization after activation by thrombin relative to control aged mice [111]. The aged platelet-deficient SOD2 mice also had an increase in carotid artery thrombosis in the Rose Bengal phytochemical injury model [111]. The hyperreactive platelet phenotype from aged platelet-deficient SOD2 mice was rescued by a SOD2 mimetic compound GC4419 suggesting that this approach could be explored in other oxidant stress-induced thrombotic states.

### 3.3. Thiol Isomerases Convert Redox Cues to a Thrombotic Response

Src family kinases and phosphatases are not the only cysteine sensors converting the redox environment to a prothrombotic response. We recently found that thiol isomerases also participate in the thrombotic potential of oxLDL [112]. The thrombogenicity of intravenous injection of oxLDL was demonstrated by multiple independent laboratories [72,91,113]. Intravenous injection of oxLDL promotes thrombosis and increases pro-coagulant pathways through mechanisms that are yet to be defined [91,114]. OxLDL itself contains lipids and amino acid hydroperoxides [115] that we hypothesize could promote thrombosis through cysteine modification [24].

Thiol isomerases are a family of 21 oxidoreductase proteins that are found in the endoplasmic reticulum to promote disulfide formation and rearrangement. They are essential for protein folding and are thus essential to life. Thiol isomerases contain thioredoxin-like domains that contain a CXXC motif within their catalytic site for mediating oxidative protein folding [25]. Cysteines within the CXXC motif are among the most susceptible to electrophilic attack by oxidants and are some of the most reactive cysteines in the entire proteome [116]. In specific conditions where cells are activated, thiol isomerases escape the endoplasmic reticulum into the extracellular milieu and mediate disulfide chemistry in their non-native environment [117,118]. The mechanisms by which thiol isomerases escape the endoplasmic reticulum despite their KDEL endoplasmic reticulum retention sequence is currently an active area of research.

Protein disulfide isomerase (PDI) is the founding member of the thiol isomerase family. It contains four thioredoxin-like domains configured structurally in an **a-b-b′-a′** sequence. The active site CGHC motifs are within the **a** and **a′** domains, whereas the hydrophobic **b** and **b′** domains function in substrate recognition. A 15 amino acid linker flanked by the **b′** and **a′** domains affords structural flexibility for the protein’s activity. An important role for PDI in thrombosis is supported by pharmacologic inhibitor studies [119,120] and targeted genetic deletion in platelets and endothelial cells [118,121,122]. Specifically, mice with conditional knockout of PDI in platelets showed a defect in thrombus formation in cremaster arterioles in the laser injury thrombosis model [117,122]. The defect was rescued by infusion of recombinant wildtype PDI but not a catalytically inactive PDI where the active site cysteines had been mutated to alanine [122]. Mutation studies of the active site cysteines in the **a** or **a′** domain revealed that the **a′** domain is important for controlling the thrombotic machinery, whereas the **a** domain is more important for cell survival [123]. The redox control of PDI’s active site cysteines is multifaceted and governed by the structure of the protein, the local environment, and the redox potential [24]. Given the susceptibility of the active site cysteines to redox reactions, it is unclear whether PDI is reduced or oxidized in thrombosis and hemostasis.

Many substrates have been identified for PDI and are reviewed in [20,25]. Biochemical studies on these substrates revealed that PDI reduces allosteric disulfide bonds, thus influencing their functions [20]. In this regard, the active site cysteines of PDI are in the reduced redox state prior to its reductase activity (breaking disulfide bonds). Other evidence suggests that PDI oxidase activity promotes disulfide bond formation on a substrate, indicating that rather than being in a reduced form, the active cysteines are in an oxidized state prior to its oxidase activity [20]. Although there is a large body of evidence indicating that PDI is important for thrombosis and is a potential antithrombotic target, we still do not know how PDI functions within the thrombotic machinery.

**Figure 4:**
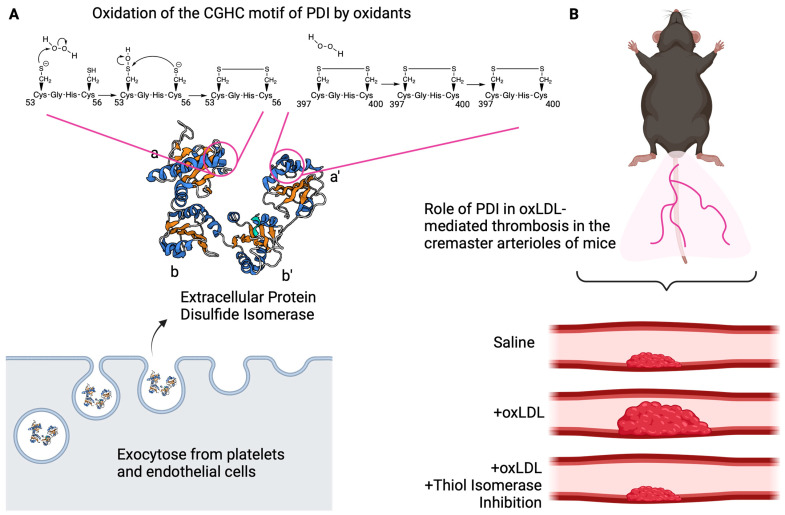
Thiol isomerases link oxidative stress to thrombosis. (**A**) Protein disulfide isomerase (PDI) contains redox-active cysteines within CGHC motifs of the catalytic **a** and **a′** domains. The CGHC motif of the **a** domain is sensitive to oxidation (sulfenylation) by peroxides, while the **a′** domain is in an oxidized state and is less sensitive. PDI secreted from activated human vein endothelial cells and platelets contains a fraction of PDI that is sulfenylated. The mechanism of sulfenylation in the CGHC motif is drawn based on the more nucleophilic N-terminal cysteine; it is not clear which cysteine is being sulfenylated by peroxides; and it is also not clear how much of the fraction of exocytosed PDI is in the reduced or oxidized state. (**B**) In a laser-injury mouse model of cremaster arteriolar thrombosis, oxLDL infusion results in increased platelet accumulation compared to saline infusion. Inhibition of thiol isomerases with a PDI blocking monoclonal antibody RL90 prevents the enhanced thrombosis observed with oxLDL. Created with BioRender.com and accessed on 4 January 2024.

Our recent findings indicate that extracellular PDI senses the redox environment and links oxidative stress to thrombosis through cysteine sulfenylation [112]. Using biochemical assays and in vitro cell biology approaches, we found that the active site cysteines of PDI are sulfenylated by peroxides (Figure 4A). Efficient sulfenylation of PDI is selective to the **a** domain and requires components of the **b** and **b′x** domains. Sulfenylation of the **a** domain is an intermediate cysteine oxoform to the disulfide. Mass spectrometry-based experiments revealed that the **a′** domain was already in an oxidized state (~80% oxidized), and that it is the **a** domain that is susceptible to oxidation. Disulfides on PDI are transferred to a ribonuclease (RNAse) that has been chemically reduced and denatured. The transfer of disulfides from PDI to RNAse accelerates the folding of RNAse into a functional enzyme in order to hydrolyze cCMP to CMP [124]. The enzymatic activity of RNAse is linked to PDI oxidase activity, where the dependency on sulfenic acid was tested with arsenite. Arsenite is a potentially selective but crude reagent to reduce sulfenic acid to free thiol [15,125,126], and using the agent prevented the enzyme-coupled refolding of RNAse by oxidized PDI.

Our hypothesis that PDI could become oxidized and contribute to thrombosis was tested ex vivo in cells and in vivo in mice. The first question was whether sulfenylated PDI is part of the pool of PDI secreted from activated cells. Platelets and endothelial cells were chosen because these are the cell types that have been shown to exocytose PDI [121,122]. Consistent with our hypothesis, we found that activating human umbilical vein endothelial cells with thrombin or platelets with SFLLRN, a protease activated receptor agonist, increased sulfenylation of exocytosed PDI (Figure 4A), while the intracellular pool of PDI did not appear to be sulfenylated. Gaspar and Laurindo hypothesized that compartmentalization of PDI within the cells could insulate the protein from being oxidized [127]. In addition, structural components of the **b** and **b′x** domains may insulate the protein once it is sulfenylated, allowing for sulfenylation to be detected within the **a** domain. Furthermore, it is likely that sulfenylation of PDI may occur in only a subset of the fraction of oxidized PDI released from the cells. The stability of the sulfenic acid is competitive between the rate of cysteine oxidation by peroxides (~10–20 M^−1^ s^−1^) [15], the rate of the probe’s interaction with sulfenic acid (~10–1700 M^−1^ s^−1^ [128]; see Section 4), and the rate of the CXXC motif transitioning to the disulfide (~10^4^–10^6^ M^−1^ s^−1^) [129]. These kinetic considerations within the context of PDI oxidation and sulfenic acid detection are yet to be tested. Nonetheless, the in vivo relevance of PDI sulfenylation in thrombosis was determined with a model of dyslipidemia, where wildtype C57Bl/6J mice were infused intravenously with oxLDL [72,113,114]. Direct infusion of oxLDL is an attempt to replicate conditions observed with atherosclerotic plaque rupture where micromolar levels of oxidized lipids are delivered to the local vascular environment. Intravenous infusion of recombinant PDI following oxLDL infusion caused sulfenylation of the enzyme, suggesting that sulfenylation of PDI is present in the pool of circulating PDI. Infusion of recombinant PDI was necessary in this study because endogenous circulating levels were below the level of detection. The thrombogenicity of intravenously injecting oxLDL was apparent by observing an increase in platelet accumulation after laser injury to the arteriole walls of the cremaster muscle. This increased platelet accumulation was prevented by limiting PDI oxidoreductase activity with RL90, an inhibitory monoclonal antibody [130,131]. These studies suggest that thiol isomerases participate in the prothrombotic phenotype in dyslipidemia possibly related to their oxidized state (Figure 4B). Although we have not yet linked PDI oxidation in vivo to the CD36 signaling pathway, such an experiment could suggest PDI as a therapeutic signaling node within the CD36 pathway for atherothrombosis. In addition, we do not know in vivo whether it is lipid hydroperoxide on oxLDL or peroxides that are generated from NADPH oxidase or other sources for PDI sulfenylation. Further sensitive methods to identify sulfenylation of endogenous circulating PDI is required. It is possible that the development of a sensitive enzyme-linked immunosorbent assay (ELISA) method or with mass spectrometry may help in determining whether endogenous circulating PDI is sulfenylated. Although typically used as a PDI oxidoreductase blocking antibody in vitro and in vivo, the RL90 antibody has been shown to inhibit other thiol isomerases (e.g., ERp57) [132]. It would be useful to further characterize existing and future thiol isomerase inhibitors to further dissect the mechanisms of each member in thrombosis.

## 4. Targeting Oxidative Cysteine Modification with Carbon Nucleophiles

**Figure 5:**
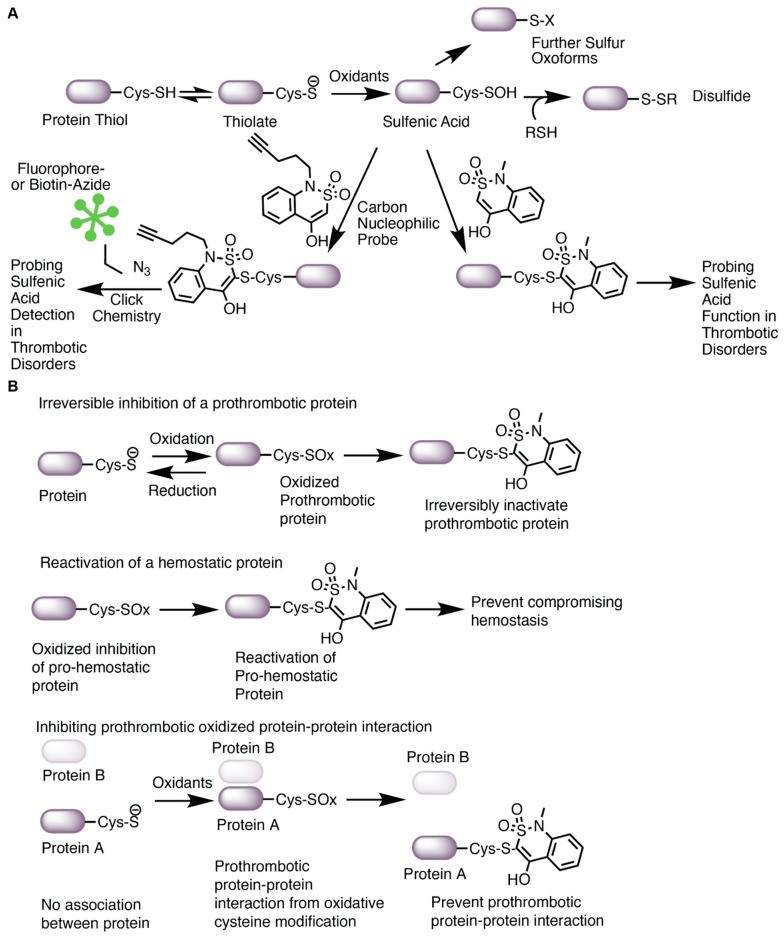
Carbon nucleophiles target oxidized cysteines. (**A**) Oxidation of the thiolate anion of cysteine promotes sulfenic acid formation, a precursor to other cysteine oxoforms. Sulfenic acids are conveniently labeled with carbon nucleophiles. In this figure, the benzothiazine-based carbon nucleophile was drawn as an example. The nucleophile modified with an alkyne arm can be adapted for click chemistry with biotin or a fluorophore to enable sulfenic acid detection by imaging or blotting. The nucleophile can also be used to assay the function of sulfenic acids in protein or cellular assays. (**B**) Covalent targeting of an oxidized cysteine could potentially serve several functions in thrombosis and hemostasis; thus, it is difficult to understand the mechanism of targeting oxidized cysteines. These hypothesized mechanisms are based on the impact of sulfenic acid-selective nucleophiles recently published by Fu et al. [137] and highlighted in a commentary [139]. Oxidation of a protein could be prothrombotic. In this case, covalent targeting of an oxidized cysteine with a nucleophile would prevent reduction of the cysteine to the thiol, thus irreversibly inactivating the prothrombotic protein. If a protein is important for hemostasis and oxidation inactivates the pro-hemostatic function of the protein, covalent labeling of the oxidized cysteine by a nucleophile would potentially re-activate the protein thus alleviating issues with preventing hemostasis. Lastly, a prothrombotic protein–protein interaction (either allosterically or covalently through e.g., disulfides) could be prevented with nucleophiles, thus limiting thrombosis. These hypothesized mechanisms are yet to be tested in vivo and will require higher selectivity of the compounds to a specific protein as well as chemoproteomic approaches to understand what other proteins are ligated.

Several molecules of natural or synthetic origin with antioxidative properties have shown potential therapeutic benefit in preventing oxidative stress-driven diseases. Yet interventions in humans have shown limited utility [105]. Many reasons may contribute to this lack of efficacy, including the challenges associated with increasing antioxidant defense mechanisms, the inability to properly scavenge specific oxidants, the bioavailability of natural antioxidants (e.g., flavonoids and other polyphenols), and the inability to target specific cells and tissues (all reviewed in [105]). One potential approach to overcoming these obstacles is to understand the targets of oxidants and the functional consequences of the oxidation. Oxidation of cysteine is one of the best models for targeted therapy given the available chemical biology tools for cysteine redox forms [133]. As discussed in Section 2, cysteine is oxidized to many sulfur species. The sulfenic acid oxoform is conveniently labeled by carbon nucleophiles [15,134]. Selective targeting with the nucleophiles serves two functions, as shown in Figure 5A: (1) it prevents further oxidation or reduction back to a free thiol as it covalently forms a thioether bond, allowing for the assaying of sulfenic acid function; and (2) coupled with azide–alkyne cycloaddition chemistry, commonly known as “click” chemistry, the nucleophiles provide a way to identify the protein target and site of oxidation [13] using fluorophores or other adducts. In this section, we discuss recent advances with a nucleophilic chemical library to characterize the sulfenome within cells and to show that the nucleophilic “tone” of the fragments could be leveraged to control the function of the oxidized protein.

In covalent ligand discovery, expanding a nucleophile library to target oxidized cysteines while not effecting the reduced state is at an early stage of development. Covalent targeting of oncogenic drivers (e.g., EGFR, KRAS) is an approach for the therapeutic treatment for cancer [135], although not in the context of oxidative modification. Oxidation of a cysteine to a sulfenic acid makes it electrophilic, a physicochemical property that can be leveraged for site directed labeling [15]. Using condensation reactions first studied in 1974 [136], the Carroll lab adapted the dimedone-based (1,3-dimethyl-1,3-cyclohexanedione; DYn-2) probe to label sulfenic acid (second order rate constants of 10 M^−1^ s^−1^) [38,128]. Later, faster-reacting nucleophiles were identified that target different sulfenylated cysteines, with a benzothiazine-based probe having the fastest kinetics (1700 M^−1^ s^−1^) [128]. Using this probe coupled to tandem mass spectrometry for global analysis, this team identified 622 sulfenic acid sites on 477 proteins in the proteome of adenocarcinoma cell lines [137]. They also synthesized 65 nucleophilic analogs [137] of an electrophilic cysteine-targetable library [138] and found that selective nucleophiles ligated reactive sulfenic acids differently when comparing the sulfenome versus the cysteinome [137]. Functionally, these nucleophilic covalent fragments impacted cellular activity. Specifically, oxidation of glyceraldehyde-3-phosphate dehydrogenase (GAPDH), glutathione *S*-transferase omega (GSTO1), and acetyl-CoA acyltransferase 1 (ACAT1) inhibited the enzymes, and the labeling with nucleophiles prevented the reversibility to a free thiol for enzyme reactivation. In another experiment, oxidation of the antioxidant peroxiredoxin-like 2A (PRXL2A) enzyme prevented its regulation of MAP kinase signaling; carbon nucleophiles targeting the oxidized cysteine of PRXL2A reactivated MAP kinase signaling. Protein–protein interaction was also investigated with heparin-binding growth factor (HDGF) interacting with nucleolin. Oxidation of HDGF and labeling with the nucleophile prevented the interaction. Lastly, BRCA2 and p21-interacting DNA repair protein (BCCIP)-mediated nucleotide damage was investigated linking oxidation events to the potential health of the cell through regulating genome instability. Covalent labeling of the functional cysteine that was oxidized in BCCIP decreased DNA damage repair by preventing p21 binding. These studies highlight specific approaches with a library of nucleophiles that can be used to identify sites of oxidation, determine the functional relevance of the oxidized sites, and suggest whether these sites could be targeted by specific nucleophiles for drug development [137,139].

In our studies, we used carbon nucleophiles to detect sulfenylation (as described in Section 3.2 and Section 3.3) and to assess the function of cysteine sulfenylation in oxidative stress-related thrombosis. Using probes [128] that differentially label sulfenic acids at different kinetic rates, we studied the effect of sulfenylation on platelet aggregatory and procoagulant activity induced by oxLDL/CD36 signaling [91,140]. We found that oxLDL enhanced platelet aggregation and phosphatidylserine externalization compared to control [91,92], and that a benzothiazine-based probe prevented oxLDL-induced platelet aggregation with an IC_50_ of 2 mM compared to 1,3-cyclohexanedione (5.9 mM), pyrrolidine (9.2 mM), and piperidine-based probes (10.2 mM) [91]. Importantly, the benzothiazine probe did not impact platelet aggregation mediated by the P2Y1/12 receptor agonist ADP or the GPVI receptor agonist collagen-related peptide [91]. In these experiments, platelet procoagulant activity mediated by the externalization of negatively charged phosphatidylserine (PS) [141] occurred after stimulating platelets with oxLDL in the presence of a GPVI receptor agonist [92,114] and was also impacted by protein sulfenylation as benzothiazine and 1,3-cyclohexanedione at the IC_50_ concentrations prevented the oxLDL-mediated increase in PS externalization [91]. These studies suggest that sulfenylation of proteins within platelet activation pathways in conditions simulating hyperlipidemic redox stress is relevant to thrombosis.

The connection between sulfenylation and oxidative stress-related thrombosis was further evaluated in vivo [91]. Wildtype C57Bl/6 mice fed a high fat/high cholesterol diet to elevate circulating lipoproteins to levels sufficient to induce platelet hypersensitivity [86,91,113] were injected intraperitoneally with increasing concentration of the benzothiazine-based probe (from 25 mg/kg up to 100 mg/kg) prior to initiating carotid artery injury by topical application of ferric chloride. The high fat diet-fed mice showed decreased time to cessation of arterial blood flow relative to control diet (indicative of a hyperreactive platelet phenotype) and this was normalized back to that seen in the control diet conditions with the 25 mg/kg dose of benzothiazine. Increasing the concentration of benzothiazine further increased the time to blood flow cessation. These studies were repeated in a second thrombosis model induced by laser injury to the cremaster arterioles after infusion of oxLDL. Similar to the findings with the ferric chloride model, platelet and fibrin deposition after laser injury were increased in the presence of oxLDL and this was prevented with 25 mg/kg benzothiazine. The probe had no impact in control mice infused with saline instead of oxLDL. These studies underscore the relevance of sulfenylation in oxidative stress thrombosis and suggest the possibility that targeted covalent inhibition with nucleophiles might be useful in disease conditions.

Covalent ligation of sulfenylated cysteines in thrombosis requires much more investigation. Ligating an oxidized cysteine could manifest into multiple mechanisms, as found in the study by Fu et al. [137]. Covalent ligation of an oxidized cysteine could inhibit a protein by irreversibly preventing reduction of the cysteine to a free thiol. In this context, it could be a way to inhibit a protein that is only activated in diseased conditions and thus does not impact hemostasis (Figure 5B, top). What would be the proteins that promote thrombosis in diseased conditions but are not essential for hemostasis? In another context, covalent ligation of the oxidized cysteine allows for re-activation of a protein (Figure 5B, middle). If a protein is pro-hemostatic and oxidation “kills” the protein, could covalent targeting of the oxidized cysteine re-activate the protein’s hemostatic function during oxidative stress? This might be relevant in conditions where efficient antithrombotic regiments increase the risk for bleeding complication or in coagulopathy where bleeding is present. An example of this may be observed during the inflammation of disseminated intravascular coagulation. In this condition, clotting factors and platelets are “used up” in the later stage of the disease, increasing the risk for bleeding complications. Reactivation of an oxidized pro-hemostatic protein might alleviate the risk for bleeding. Lastly, targeting oxidized cysteines within a prothrombotic protein–protein interaction complex may prevent thrombosis while maintaining hemostasis (Figure 5B, bottom). CD36 is a great example of this; it is highly present in lipid rafts and coordinates with other membrane proteins, including tetraspanins [142], NaK-ATPase [143], toll like receptors [144], and integrins [145], for pro-atherogenic and pro-thrombotic signaling. Preventing such interactions allosterically through targeting oxidized cysteines important for protein–protein interaction could alleviate athero progression and subsequent thrombosis. The current limitation is the off-target effects observed with covalent ligation. The recent data from Julio et al. suggest that covalent targeting of cysteines could trigger a stress response within cells, causing proteins to aggregate, and increase proteasomal degradation [146], supporting the indispensable need to address the issue of selectivity. The structure of a lead nucleophilic fragment could be refined to target a specific protein of interest through traditional structure activity relationship coupled with biochemical validation (e.g., molecular modeling and simulations, in vitro protein assays). Further characterization of whether ligating the oxidized protein of interest impacts the protein’s function and subsequent cellular activity would help in transitioning lead nucleophiles into animal thrombosis models and pre-clinical studies.

Overall, the proof-of-principles in the nucleophilic fragment screen from the Carroll lab, as well as our in vivo functional thrombosis studies in dyslipidemia with carbon nucleophiles, provides an opportunity for covalently targeting cysteine oxidation. A nucleophilic fragment screening in platelets, endothelial cells, immune cells, and red cells during oxidative stress coupled to global chemoproteomic platforms would be essential in understanding the sulfenome that controls the thrombotic machinery. Initial efforts have already been investigated for the sulfenome in pathogen-inactivated platelets [147], which identified many proteins of the cytoskeletal and integrin pathways that are cysteine oxidized. Although we found that the probe prevented thrombosis in dyslipidemia, further characterization of the compound in vivo is required.

## 5. Conclusions

Many antioxidative therapies have shown limited utility in decreasing the risk of cardiovascular events. One potential approach is to understand the target and the functional consequence of oxidation. Cysteine oxidation is a type of oxidative posttranslational modification present during oxidative stress. While pathologic conditions are associated with oxidative stress, strategies to target oxidative cysteine modification could be exploited to prevent thrombosis in diseases while not impacting hemostasis. This could be achieved by taking advantage of the physicochemistry of cysteines using chemical biology. The studies outlined in this review suggests that oxidative cysteine modification is important in regulating thrombosis. Specifically, the CD36 signaling pathway in atherothrombosis is a system where the redox environment is translated into a selective phosphorylation-driven prothrombotic response. In addition, thiol isomerases have highly reactive cysteines that are sulfenylated. Thiol isomerases thus convert the promiscuous redox cues into selective disulfide signaling, a different cysteine oxoform that is substrate-dependent for thrombosis. As carbon nucleophiles covalently label sulfenylation, they are useful tools for probing cysteine oxidation in model systems and could be used as future backbones for drug development against oxidized thiols.

## Data Availability

No new data were created or analyzed in this study. Data sharing is not applicable to this article.
